# Natural Reassortment of Eurasian Avian-Like Swine H1N1 and Avian H9N2 Influenza Viruses in Pigs, China

**DOI:** 10.3201/eid2807.220642

**Published:** 2022-07

**Authors:** Wanying Sun, Samuel S.M. Cheng, Kristy N.T. Lam, Tsz C. Kwan, Ricky W.K. Wong, Leo H.K. Lau, Gigi Y.Z. Liu, Leo L.H. Luk, John K.C. Li, Haogao Gu, Malik Peiris, Leo L.M. Poon

**Affiliations:** School of Public Health, The University of Hong Kong, Hong Kong, China (W. Sun, S.S.M. Cheng, K.N.T. Lam, T.C. Kwan, R.W.K. Wong, L.H.K. Lau. G.Y.Z. Liu, L.L.H. Luk, J.K.C. Li, H. Gu, M. Peiris, L.L.M. Poon);; Centre for Immunology and Infection, Hong Kong (M. Peiris, L.L.M. Poon);; HKU-Pasteur Research Pole, The University of Hong Kong, Hong Kong (M. Peiris, L.L.M. Poon)

**Keywords:** influenza, Eurasian avian-like swine H1N1 virus, avian H9N2 virus, zoonotic influenza A viruses, pigs, China, viruses, zoonoses

## Abstract

Several zoonotic influenza A viruses detected in humans contain genes derived from avian H9N2 subtypes. We uncovered a Eurasian avian-like H1N1 swine influenza virus with polymerase basic 1 and matrix gene segments derived from the H9N2 subtype, suggesting that H9N2 viruses are infecting pigs and reassorting with swine influenza viruses in China.

Swine are regarded as a mixing vessel for influenza A viruses (IAVs) (*1*). Avian, swine, and human IAVs can co-infect pigs and generate novel reassortants of zoonotic or pandemic potential. The emergence of pandemic H1N1 IAV (pH1N1), containing viral segments from avian, swine, and human viruses, highlighted the key role of pigs in contributing to IAV reassortment and evolution (*2*). Research in China also showed evidence of avian H5, H7, H9, and H10 influenza infections in pigs (*3*). Avian IAVs linked to human infection in this region contained internal genes derived from avian H9N2 viruses, indicating that the internal genes of the H9N2 virus might aid zoonotic transmission (*4*). We report detection of a swine IAV with polymerase basic (PB) 1 and matrix (M) gene segments of avian H9N2 origin.

In April 2021, we resumed monthly influenza surveillance program of imported pigs in a local slaughterhouse, which had been interrupted by COVID-19 outbreaks (*5*). We collected individual nasal swab samples (≈75 samples per visit), which we kept chilled in virus transport medium until they reached the laboratory. We then subjected swab samples to IAV isolation by using MDCK cells, as previously described (*2*). We identified cultures with cytopathic effect and tested them using a standard hemagglutination assay with turkey red blood cells. We tested hemagglutination-positive cultures with a universal influenza reverse transcription PCR assay specific for M segments (*6*). We studied samples that were positive for this reaction by using next-generation sequencing to deduce the full virus genomes (*6*).

During April 2021–February 2022, we collected a total of 829 porcine nasal swab samples (Table). We isolated 8 IAVs: 7 from August 2021 and 1 from September 2021. Virus sequences deduced from this study are available from GISAID (isolate nos. EPI_ISL_12471293–300). We compared those sequences with reference sequences (Appendix Table). IAVs detected in August 2021 were H3N2 viruses. The hemagglutinin (HA) and neuraminidase (NA) segments of those viruses were associated with human-like H3N2 swine influenza A virus; however, their internal gene segments all were derived from the pH1N1 lineage (Figure; Appendix Figures 1–6). These viruses were genetically not identical but highly similar. The influenza-positive pigs came from farms located in 2 provinces across southern China. Because this slaughterhouse followed a daily clearance policy requiring that all imported live pigs be slaughtered within 24 hours of admittance, our results suggest influenza transmission between pigs in the preslaughter transport chain outside Hong Kong. This H3N2 genotype was previously detected in pigs from Guangxi, China (*7*).

**Table:** Swine influenza viruses detected in imported pigs, China, April 2021–February 2022

Year and month	No. nasal swabs	No. virus isolates	Isolation rate, %
2021			
Apr	60	0	0
May	75	0	0
Jun	75	0	0
Jul	75	0	0
Aug	75	7*	9.3
Sep	75	1†	1.3
Oct	79	0	0
Nov	85	0	0
Dec	80	0	0
2022			
Jan	75	0	0
Feb	75	0	0
Total	829	8	0.97

*All H3N2; pigs were imported from 2 provinces in southern China. †H1N1, pig was from imported from a province in southern China.

**Figure:**
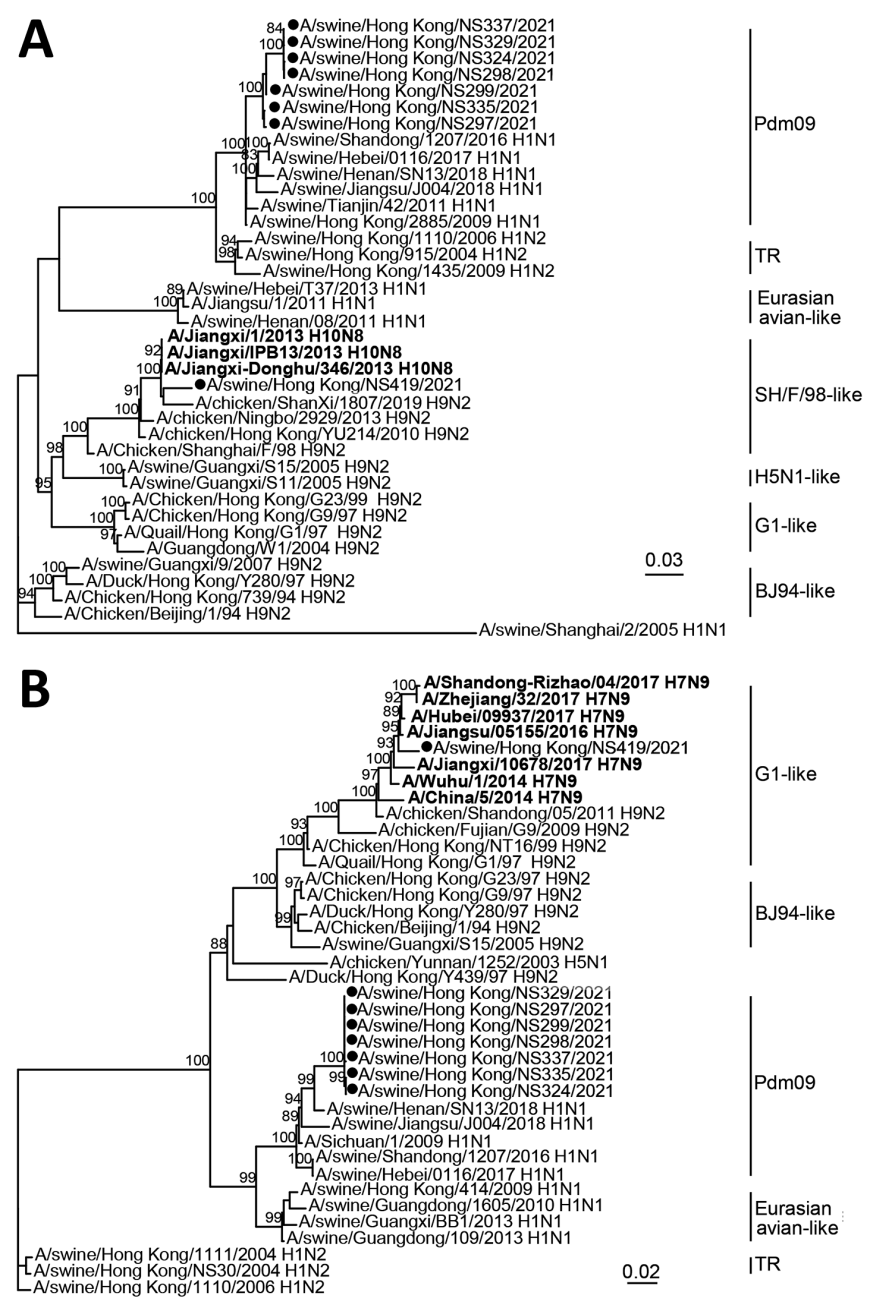
Phylogenetic tree of polymerase basic 1 (A) and matrix (B) gene sequences of swine influenza viruses from China and reference sequences. Bold indicates human H7N9 and H10N8 sequences. Viral sequences generated in this study (black circles) and those downloaded from public domains (Appendix Table) were aligned by using Muscle version 3.8 (http://www.drive5.com/muscle). Phylogenetic trees were constructed by IQ-TREE 1.6.12 (http://www.iqtree.org) by using the generalized time reversible plus gamma model. Major animal viral lineages are as shown. Bootstrap values ≥80% are shown. Scale bar indicates estimated genetic distance.

The swine H1N1 IAV that we isolated in September 2021, A/swine/HK/NS419/2021, a reassortant between multiple swine influenza lineages (Figure; Appendix Figures 1–6). The PB1 and M gene segments of this virus are of avian H9N2 virus subtype. This virus contains PB2, polymerase acidic, and NA gene segments derived from the pH1N1 lineage. Its HA and NA gene segments are of Eurasian avian-like H1N1 lineage, and its nonstructural gene segment is of a triple reassortant lineage. We further purified the isolated virus by using plaque assays to exclude the possibility of a mixed infection. We confirmed that all plaque-purified viral clones had an identical genotype.

The A/swine/HK/NS419/2021 isolate featured a PB1 gene segment of SH/F/98‐like lineage and an M gene segment of G1-like H9N2 lineage (Figure). Similar PB1 and M sequences have been detected in zoonotic viruses in humans (Figure), PB1 in H10N8 and M in H7N9, but we did not find mutations known for mammalian host adaptation in these 2 segments. The encoded proteins of the PB1 and M gene segments that we isolated featured amino acid sequences rarely observed in mammalian and avian IAVs, including H9 (PB1, 97K, 156N, 397V, 535V, 688I, and 704T; M1, 31I and 46V; and M2, 25S). We could not determine whether these were random or adaptive mutations. The PB1 segment of avian H9N2 is highly compatible to other polymerase genes from mammalian IAVs (*8*). Such results suggest the need for further characterization of these mutations, particularly those in the PB1 gene.

A recent report in China discussed multiple Eurasian avian-like H1N1 swine influenza reassortants with internal genes derived from pH1N1 and triple reassortant lineages (*9*). One group of these reassortants (genotype 4) displayed a genotype similar to A/swine/HK/NS419/2021, the only exception being that the virus’s PB1 and M gene segments were of pH1N1 lineage. That report showed that genotype 4 Eurasian avian-like swine IAVs can bind to human sialic acid receptors (i.e., α2,3), enabling efficient virus replication in human airway epithelial cells, and achieve efficient aerosol transmission in ferrets (*9*). Serologic surveillance further showed that 10% of studied swine workers were positive for the genotype 4 reassortant (*9*). Our own sequence analyses suggest that some of the genotype 4 viruses and our Eurasian avian-like H1N1 viruses might share a common ancestry (e.g., A/swine/Shandong/1207/2016; Appendix Figures 1–6). Further risk assessment on the pandemic potential of this genotype and its reassortants is needed (*10*).

In summary, many zoonotic IAVs in humans have genes derived from H9N2 subtypes. Our results suggest that avian H9N2 IAVs are infecting swine and reassorting with swine IAVs, which indicates the need for continued monitoring of swine IAVs in both China and outlying regions.

Appendix Additional information about natural reassortment of Eurasian avian-like swine H1N1 and avian H9N2 influenza viruses in pigs, China.
