# Supplementary material for: Natural Reassortment of Eurasian Avian-Like Swine H1N1 and Avian H9N2 Influenza Viruses in Pigs, China
Source: Emerg Infect Dis. 2022 Jul;28(7):1509–12. doi: 10.3201/eid2807.220642 (PMC9239857; doi:10.3201/eid2807.220642)
Supplement: Appendix — Additional information about natural reassortment of Eurasian avian-like swine H1N1 and avian H9N2 influenza viruses in pigs, China. [file 22-0642-Techapp-s1.pdf]

Article DOI: http://doi.org/10.3201/eid2807.220642

Natural Reassortment of Eurasian Avian-like Swine H1N1 and Avian H9N2 Influenza Viruses in Pigs, China

Appendix

Appendix Table. Viral sequences used in this study

Segment	Strain name	Accession number
PB2	A/swine/Fujian/204/2007	FJ536816
PB2	A/swine/Wisconsin/30954/1976	CY036806
PB2	A/swine/Shanghai/2/2005	FJ789824
PB2	A/swine/Hong Kong/915/2004	GQ229273
PB2	A/swine/Hong Kong/78/2003	GQ229313
PB2	A/swine/Hong Kong/1562/2005	GQ229337
PB2	A/swine/Hong Kong/1110/2006	GQ229377
PB2	A/swine/Hong Kong/1435/2009	CY061650
PB2	A/swine/Hong Kong/NS1583/2009	CY061722
PB2	A/swine/Hong Kong/2299/2009	CY061730
PB2	A/swine/Hong Kong/2314/2009	CY061738
PB2	A/swine/Hong Kong/NS1809/2009	CY061746
PB2	A/swine/Hong Kong/NS1810/2009	CY061754
PB2	A/swine/Hong Kong/2885/2009	CY061762
PB2	A/swine/Hong Kong/2886/2009	CY061770
PB2	A/swine/Hong Kong/2974/2009	CY061786
PB2	A/swine/Hong Kong/189/2010	CY061802
PB2	A/swine/Hong Kong/1795/1994	CY085102
PB2	A/swine/Hong Kong/8278/2001	CY085371
PB2	A/swine/Hong Kong/227/2002	CY085491
PB2	A/swine/Hong Kong/1248/2002	CY085523
PB2	A/swine/Hong Kong/1578/2003	CY085603
PB2	A/swine/Hong Kong/1111/2004	CY085659
PB2	A/swine/Hong Kong/729/2005	CY085731
PB2	A/swine/Hong Kong/72/2007	CY085811
PB2	A/swine/Hong Kong/NS252/2009	CY085995
PB2	A/swine/Guangdong/1605/2010	JN375017
PB2	A/swine/Guangdong/1623/2010	JN375023
PB2	A/swine/Shaanxi/s2/2012	JX963608
PB2	A/Jiangsu/1/2011	KF057091
PB2	A/swine/Guangdong/109/2013	KP404209
PB2	A/swine/Hunan/30/2013	KP404289
PB2	A/swine/Hong Kong/299/1993	CY084982
PB2	A/swine/Hong Kong/1223/1993	CY085054
PB2	A/swine/Hebei/0116/2017	MN416337
PB2	A/swine/Henan/SN13/2018	MN416362
PB2	A/swine/Jiangsu/J004/2018	MN416364
PB2	A/swine/Shandong/1207/2016	MN416383
PB1	A/Chicken/Hong Kong/G9/97	AF156416
PB1	A/Chicken/Hong Kong/G23/99	AF156417
PB1	A/Duck/Hong Kong/Y280/97	AF156419
PB1	A/Quail/Hong Kong/G1/97	AF156421
PB1	A/Chicken/Hong Kong/739/94	AF156422
PB1	A/Chicken/Beijing/1/94	AF156423
PB1*	A/Jiangxi-Donghu/346/2013	EPI_ISL_152846
PB1*	A/Jiangxi/1/2013	EPI_ISL_174265

Segment	Strain name	Accession number
PB1	A/Chicken/Shanghai/F/98	AY253751
PB1	A/swine/Guangxi/S11/2005	EU086330
PB1	A/swine/Guangxi/S15/2005	EU086331
PB1	A/swine/Hong Kong/915/2004	GQ229268
PB1	A/swine/Hong Kong/1110/2006	GQ229372
PB1	A/swine/Hong Kong/1435/2009	CY061651
PB1	A/swine/Hong Kong/2885/2009	CY061763
PB1	A/swine/Guangxi/9/2007	CY075044
PB1	A/Jiangsu/1/2011	KF057098
PB1	A/chicken/Hong Kong/YU214/2010	KF260616
PB1	A/Jiangxi/IPB13/2013	KJ406535
PB1	A/swine/Tianjin/42/2011	KP404402
PB1	A/chicken/Ningbo/2929/2013	KP415347
PB1	A/Guangdong/W1/2004	KX867847
PB1	A/swine/Hebei/0116/2017	MN416424
PB1	A/swine/Hebei/T37/2013	MN416433
PB1	A/swine/Henan/08/2011	MN416437
PB1	A/swine/Henan/SN13/2018	MN416449
PB1	A/swine/Shandong/1207/2016	MN416470
PB1	A/chicken/ShanXi/1807/2019	MN780833
PB1	A/swine/Shanghai/2/2005	FJ789825
PB1	A/swine/Jiangsu/J004/2018	MN416451
PA	A/swine/Fujian/204/2007	FJ536815
PA	A/swine/Wisconsin/30954/1976	CY036804
PA	A/swine/Shanghai/2/2005	FJ789826
PA	A/swine/Hong Kong/915/2004	GQ229272
PA	A/swine/Hong Kong/78/2003	GQ229312
PA	A/swine/Hong Kong/1562/2005	GQ229336
PA	A/swine/Hong Kong/1110/2006	GQ229376
PA	A/swine/Hong Kong/1435/2009	CY061652
PA	A/swine/Hong Kong/NS1583/2009	CY061724
PA	A/swine/Hong Kong/2299/2009	CY061732
PA	A/swine/Hong Kong/2314/2009	CY061740
PA	A/swine/Hong Kong/NS1809/2009	CY061748
PA	A/swine/Hong Kong/NS1810/2009	CY061756
PA	A/swine/Hong Kong/2885/2009	CY061764
PA	A/swine/Hong Kong/2886/2009	CY061772
PA	A/swine/Hong Kong/2974/2009	CY061788
PA	A/swine/Hong Kong/189/2010	CY061804
PA	A/swine/Hong Kong/299/1993	CY084984
PA	A/swine/Hong Kong/1223/1993	CY085056
PA	A/swine/Hong Kong/1795/1994	CY085104
PA	A/swine/Hong Kong/8278/2001	CY085373
PA	A/swine/Hong Kong/227/2002	CY085493
PA	A/swine/Hong Kong/1248/2002	CY085525
PA	A/swine/Hong Kong/1578/2003	CY085605
PA	A/swine/Hong Kong/1111/2004	CY085661
PA	A/swine/Hong Kong/729/2005	CY085733
PA	A/swine/Hong Kong/NS252/2009	CY085997
PA	A/swine/Guangdong/1605/2010	JN375094
PA	A/swine/Guangdong/1623/2010	JN375099
PA	A/swine/Shaanxi/s2/2012	JX963609
PA	A/Jiangsu/1/2011	KF057105
PA	A/swine/Guangdong/109/2013	KP404211
PA	A/swine/Hunan/30/2013	KP404291
PA	A/swine/Hong Kong/72/2007	CY085813
PA	A/swine/Hebei/0116/2017	MN416524
PA	A/swine/Henan/SN13/2018	MN416549
PA	A/swine/Jiangsu/J004/2018	MN416551
PA	A/swine/Shandong/1207/2016	MN416570
HA	A/swine/Shanghai/2/2005	EU502885
HA	A/swine/Hong Kong/915/2004	GQ229269
HA	A/swine/Hong Kong/78/2003	GQ229309

Segment	Strain name	Accession number
HA	A/swine/Iowa/A01049239/2010	JN162047
HA	A/swine/Guangdong/2670/2010	JN375298
HA	A/swine/Illinois/SG1141/2003	CY099191
HA	A/swine/Jiangsu/zg6/2010	JN809152
HA	A/swine/England/39572/2001	CY116151
HA	A/swine/England/57610/1999	CY116222
HA	A/swine/Sarthe/0262/2010	FR871195
HA	A/Swine/Spain/80598LP4/2007	HF674912
HA	A/swine/Tianjin/3/2011	KJ725064
HA	A/swine/Italy/287785/2012	KJ847624
HA	A/swine/Guangdong/3583/2011	KM027639
HA	A/swine/Guangdong/3676/2011	KM027655
HA	A/swine/Guangxi/2893/2011	KM028095
HA	A/swine/Guangxi/2895/2011	KM028103
HA	A/swine/Hong Kong/NS52/2012	KM029159
HA	A/swine/Guangdong/2/2013	KP404164
HA	A/swine/Guangdong/95/2013	KP404204
HA	A/swine/Spain/24237/2011	KR700161
HA	A/swine/Spain/29257/2012	KR700177
HA	A/swine/England/463180/1994	KR700601
HA	A/swine/England/000304/2009	KR700648
HA	A/swine/England/7800/2012	KR700743
HA	A/swine/Beijing/82/2011	MN416593
HA	A/swine/Hebei/0116/2017	MN416597
HA	A/swine/Henan/SN13/2018	MN416622
HA	A/swine/Jiangsu/J004/2018	MN416624
HA	A/swine/Shandong/1207/2016	MN416643
HA	A/swine/Shandong/436/2012	MN416647
HA	A/swine/Hong Kong/q066/99	AY363518
HA	A/swine/Korea/CY07/2007	EU798795
HA	A/swine/Korea/CY10/2007	EU798797
HA	A/swine/Sichuan/01/2006	EU655692
HA	A/swine/Chachoengsao/2003	AB434344
HA	A/swine/Minnesota/7931/2007	FJ519977
HA	A/swine/Pennsylvania/a01076503/2010	JQ738139
HA	A/swine/Minnesota/A01076701/2010	JQ783069
HA	A/Malaysia/29930/2004	CY118818
HA	A/swine/Guangdong/L21/2011	JX096504
HA	A/swine/Guangdong/L5/2010	JX414232
HA	A/swine/Wisconsin/H02AS8/2002	CY116566
HA	A/swine/Denmark/14348–9/2003	KC900244
HA	A/swine/Minnesota/SG1294/2007	CY158121
HA	A/swine/South Dakota/SG1453/2003	CY159113
HA	A/swine/Thailand/CU-S3474N/2012	KJ526029
HA	A/swine/Thailand/CU-S3474N/2012	KM355372
HA	A/swine/Missouri/A01476459/2012	KP137795
HA	A/swine/Iowa/A01476969/2014	KP288136
HA	A/swine/Missouri/A01840170/2014	KP461660
HA	A/swine/Missouri/A01840324/2015	KP765818
HA	A/swine/Chachoengsao/2002	AB571803
HA	A/swine/Chonburi/2002	AB573013
NP	A/swine/Fujian/204/2007	FJ536813
NP	A/swine/Wisconsin/30954/1976	CY036802
NP	A/swine/Shanghai/2/2005	FJ789828
NP	A/swine/Hong Kong/915/2004	GQ229270
NP	A/swine/Hong Kong/78/2003	GQ229310
NP	A/swine/Hong Kong/1562/2005	GQ229334
NP	A/swine/Hong Kong/1110/2006	GQ229374
NP	A/swine/Hong Kong/1435/2009	CY061654
NP	A/swine/Hong Kong/NS1583/2009	CY061726
NP	A/swine/Hong Kong/2299/2009	CY061734
NP	A/swine/Hong Kong/2314/2009	CY061742
NP	A/swine/Hong Kong/NS1809/2009	CY061750

Segment	Strain name	Accession number
NP	A/swine/Hong Kong/NS1810/2009	CY061758
NP	A/swine/Hong Kong/2885/2009	CY061766
NP	A/swine/Hong Kong/2886/2009	CY061774
NP	A/swine/Hong Kong/2974/2009	CY061790
NP	A/swine/Hong Kong/189/2010	CY061806
NP	A/swine/Hong Kong/8278/2001	CY085375
NP	A/swine/Hong Kong/227/2002	CY085495
NP	A/swine/Hong Kong/1111/2004	CY085663
NP	A/swine/Hong Kong/729/2005	CY085735
NP	A/swine/Hong Kong/72/2007	CY085815
NP	A/swine/Hong Kong/NS252/2009	CY085999
NP	A/swine/Guangdong/1605/2010	JN375161
NP	A/swine/Guangdong/1623/2010	JN375167
NP	A/swine/Shaanxi/s2/2012	JX963614
NP	A/Jiangsu/1/2011	KF057119
NP	A/swine/Guangdong/109/2013	KP404213
NP	A/swine/Hunan/30/2013	KP404293
NP	A/swine/Hong Kong/299/1993	CY084986
NP	A/swine/Hong Kong/1223/1993	CY085058
NP	A/swine/Hong Kong/1795/1994	CY085106
NP	A/swine/Hong Kong/1248/2002	CY085527
NP	A/swine/Hong Kong/1578/2003	CY085607
NP	A/swine/Hebei/0116/2017	MN418741
NP	A/swine/Henan/SN13/2018	MN418766
NP	A/swine/Jiangsu/J004/2018	MN418768
NP	A/swine/Shandong/1207/2016	MN418787
NA	A/swine/Hebei/0116/2017	MN416701
NA	A/swine/Henan/SN13/2018	MN416726
NA	A/swine/Jiangsu/J004/2018	MN416728
NA	A/swine/Shandong/1207/2016	MN416747
NA	A/swine/North Carolina/2003	EF551055
NA	A/swine/Minnesota/761/2007	FJ519964
NA	A/swine/England/1382/2010	JF290393
NA	A/swine/Hong Kong/2503/2011	JQ220550
NA	A/swine/Hong Kong/NS2439/2011	JQ220549
NA	A/swine/Guangxi/NS2783/2010	JQ220548
NA	A/swine/Pennsylvania/a01076503/2010	JQ738141
NA	A/swine/Illinois/A01047014/2010	JQ791006
NA	A/swine/Minnesota/18/2012	JX853751
NA	A/swine/Minnesota/SG1294/2007	CY158123
NA	A/swine/North Carolina/01506/2006	CY159347
NA	A/swine/North Carolina/01216/2006	CY159371
NA	A/swine/Guangxi/2803/2011	KM028057
NA	A/swine/Missouri/A01476459/2012	KP137796
NA	A/swine/Spain/24237/2011	KR700163
NA	A/swine/England/038712/2012	KR700495
NA	A/swine/England/101878/2010	KR700665
NA	A/swine/England/142162/2010	KR700673
NA	A/swine/England/7800/2012	KR700745
NA	A/swine/England/072512/2013	KR701067
NA	A/swine/Guangdong/611/2006	GU086043
NA	A/swine/Iowa/H03G1/2003	GU135912
NA	A/swine/Hong Kong/NS1809/2009	CY061751
NA	A/swine/Hong Kong/2995/2009	CY061799
NA	A/swine/Hong Kong/NS1170/2004	CY085688
NA	A/swine/Hong Kong/294/2003	CY086546
NA	A/swine/Hong Kong/NS1160/2004	CY086752
NA	A/swine/Minnesota/6998/2003	CY098486
NA	A/swine/Shandong/811/2009	JQ695889
NA	A/swine/Colombia/2/2011	KC703346
NA	A/swine/North Carolina/SG1463/2004	CY159195
NA	A/swine/North Carolina/SG1172/2003	CY157665
NA	A/swine/Guangdong/2417/2010	KM027457

Segment	Strain name	Accession number
NA	A/swine/Guangdong/NS909/2012	KM027889
NA	A/swine/Hong Kong/3904/2011	KM028817
NA	A/swine/Hong Kong/973/2010	KM029513
NA	A/swine/Kagoshima/65/2012	AB911619
NA	A/swine/Hunan/196/2011	KP404286
NA	A/swine/Sichuan/62/2012	KP404382
NA	A/swine/Zhucheng/90/2014	KX264371
NA	A/swine/Hong Kong/2422/1994	CY087058
NA	A/canine/Guangxi/WZ2/2013	MG254094
NA	A/swine/Saskatchewan/D0515/2017	MK462308
NA	A/swine/Tianjin/960/2012	MN416762
NA	A/swine/Chachoengsao/2003	AB434346
NA	A/swine/Chachoengsao/2002	AB571805
NA	A/swine/Chonburi/2002	AB573015
M*	A/China/5/2014_H7N9	EPI_ISL_368803
M*	A/Hubei/09937/2017_H7N9	EPI_ISL_285591
M*	A/Jiangsu/05155/2016_H7N9	EPI_ISL_285559
M*	A/Jiangxi/10678/2017_H7N9	EPI_ISL_285246
M*	A/Shandong-Rizhao/04/2017_H7N9	EPI_ISL_285268
M*	A/wuhu/1/2014_H7N9	EPI_ISL_399682
M*	A/Zhejiang/32/2017_H7N9	EPI_ISL_285320
M	A/Chicken/Hong Kong/G9/97	AF156458
M	A/Chicken/Hong Kong/G23/97	AF156459
M	A/Duck/Hong Kong/Y280/97	AF156461
M	A/Duck/Hong Kong/Y439/97	AF156462
M	A/Quail/Hong Kong/G1/97	AF156463
M	A/Chicken/Beijing/1/94	AF156466
M	A/Chicken/Hong Kong/NT16/99	AF222664
M	A/swine/Guangxi/S15/2005	EU086321
M	A/chicken/Yunnan/1252/2003	CY029394
M	A/Sichuan/1/2009	GQ166229
M	A/swine/Hong Kong/1110/2006	GQ229375
M	A/swine/Hong Kong/NS30/2004	CY085625
M	A/swine/Hong Kong/1111/2004	CY085665
M	A/swine/Guangdong/1605/2010	JN375233
M	A/chicken/Fujian/G9/2009	JN869519
M	A/swine/Guangxi/BB1/2013	KJ174948
M	A/chicken/Shandong/05/2011	KC821204
M	A/swine/Guangdong/109/2013	KP404215
M	A/swine/Hong Kong/414/2009	CY085993
M	A/swine/Hebei/0116/2017	MN418821
M	A/swine/Henan/SN13/2018	MN418846
M	A/swine/Jiangsu/J004/2018	MN418848
M	A/swine/Shandong/1207/2016	MN418867
NS	A/swine/Guangxi/13/2006	EU015988
NS	A/swine/Fujian/204/2007	FJ536814
NS	A/swine/Beijing/26/2008	FJ536774
NS	A/swine/Wisconsin/30954/1976	CY036803
NS	A/swine/Shanghai/2/2005	FJ789837
NS	A/swine/Hong Kong/915/2004	GQ229267
NS	A/swine/Hong Kong/1562/2005	GQ229331
NS	A/swine/Hong Kong/1110/2006	GQ229371
NS	A/swine/Guangdong/628/2006	GU086053
NS	A/swine/Hong Kong/1435/2009	CY061657
NS	A/swine/Hong Kong/NS1583/2009	CY061729
NS	A/swine/Hong Kong/2299/2009	CY061737
NS	A/swine/Hong Kong/2314/2009	CY061745
NS	A/swine/Hong Kong/NS1809/2009	CY061753
NS	A/swine/Hong Kong/NS1810/2009	CY061761
NS	A/swine/Hong Kong/2885/2009	CY061769
NS	A/swine/Hong Kong/2886/2009	CY061777
NS	A/swine/Hong Kong/2974/2009	CY061793
NS	A/swine/Hong Kong/189/2010	CY061809

Segment	Strain name	Accession number
NS	A/swine/Guangdong/1/2010	HM189304
NS	A/swine/Guangdong/02/2008	HM223598
NS	A/swine/Guangdong/1/2010	HQ853346
NS	A/swine/Hong Kong/227/2002	CY085498
NS	A/swine/Hong Kong/1248/2002	CY085530
NS	A/swine/Hong Kong/1578/2003	CY085610
NS	A/swine/Hong Kong/729/2005	CY085738
NS	A/swine/Hong Kong/NS252/2009	CY086002
NS	A/swine/Hubei/01/2009	CY091741
NS	A/swine/Hubei/03/2009	CY091749
NS	A/swine/Hubei/104/2009	CY091773
NS	A/swine/Guangdong/275/2010	JN375253
NS	A/swine/Guangdong/278/2010	JN375254
NS	A/swine/Guangdong/297/2010	JN375257
NS	A/swine/Guangdong/1437/2010	JN375267
NS	A/swine/Guangdong/1604/2010	JN375269
NS	A/swine/Guangdong/1611/2010	JN375271
NS	A/swine/Shaanxi/s2/2012	JX963611
NS	A/Jiangsu/1/2011	KF057140
NS	A/swine/Guangdong/109/2013	KP404216
NS	A/swine/Hunan/30/2013	KP404296
NS	A/swine/Hong Kong/299/1993	CY084989
NS	A/swine/Hong Kong/1223/1993	CY085061
NS	A/swine/Hong Kong/1795/1994	CY085109
NS	A/swine/Hong Kong/8278/2001	CY085378
NS	A/swine/Hong Kong/1111/2004	CY085666
NS	A/swine/Hebei/0116/2017	MN418667
NS	A/swine/Henan/SN13/2018	MN418692
NS	A/swine/Jiangsu/J004/2018	MN418694
NS	A/swine/Shandong/1207/2016	MN418713

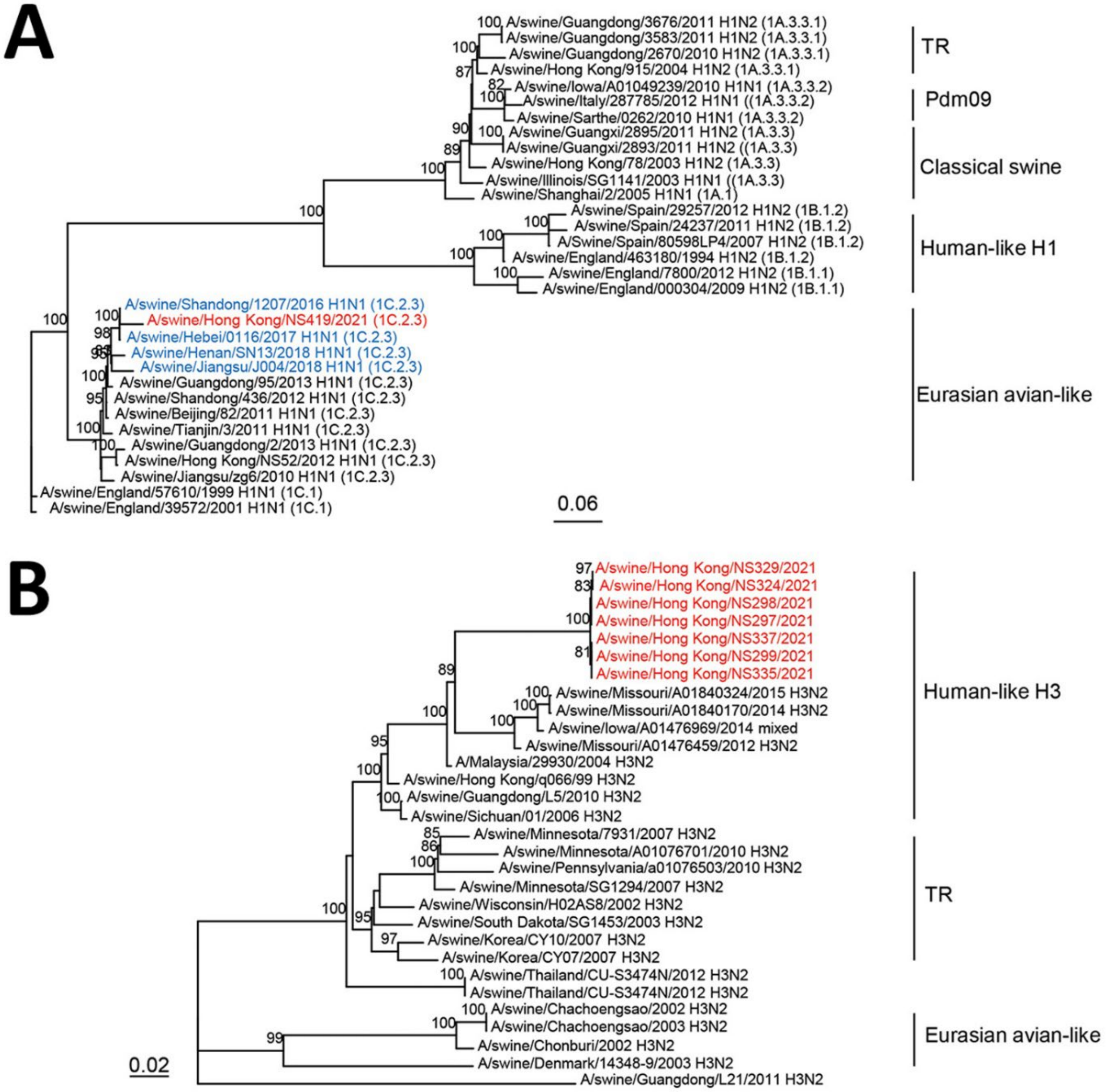

Appendix Figure 1. Phylogenetic tree of H1 (A) or H3 (B) gene sequences. Viral sequences generated from this study (red) and those downloaded from public domains (Appendix Table) were aligned using muscle v3.8 (http://www.drive5.com/muscle/). Virus sequences from G4 swine viruses (Main text) are highlighted in blue. Phylogenetic trees were then constructed by IQ-tree v1.6.12 (http://www.iqtree.org/release/v1.6.12) using the GTR+G model. Major animal viral lineages are as shown. The phylogenetic clades of the studied H1 sequences are shown in brackets (Anderson et al., mSphere 2016; https://doi.org/10.1128/mSphere.00275-16). Bootstrap values ≥80% are shown. Scale bar indicates estimated genetic distance.

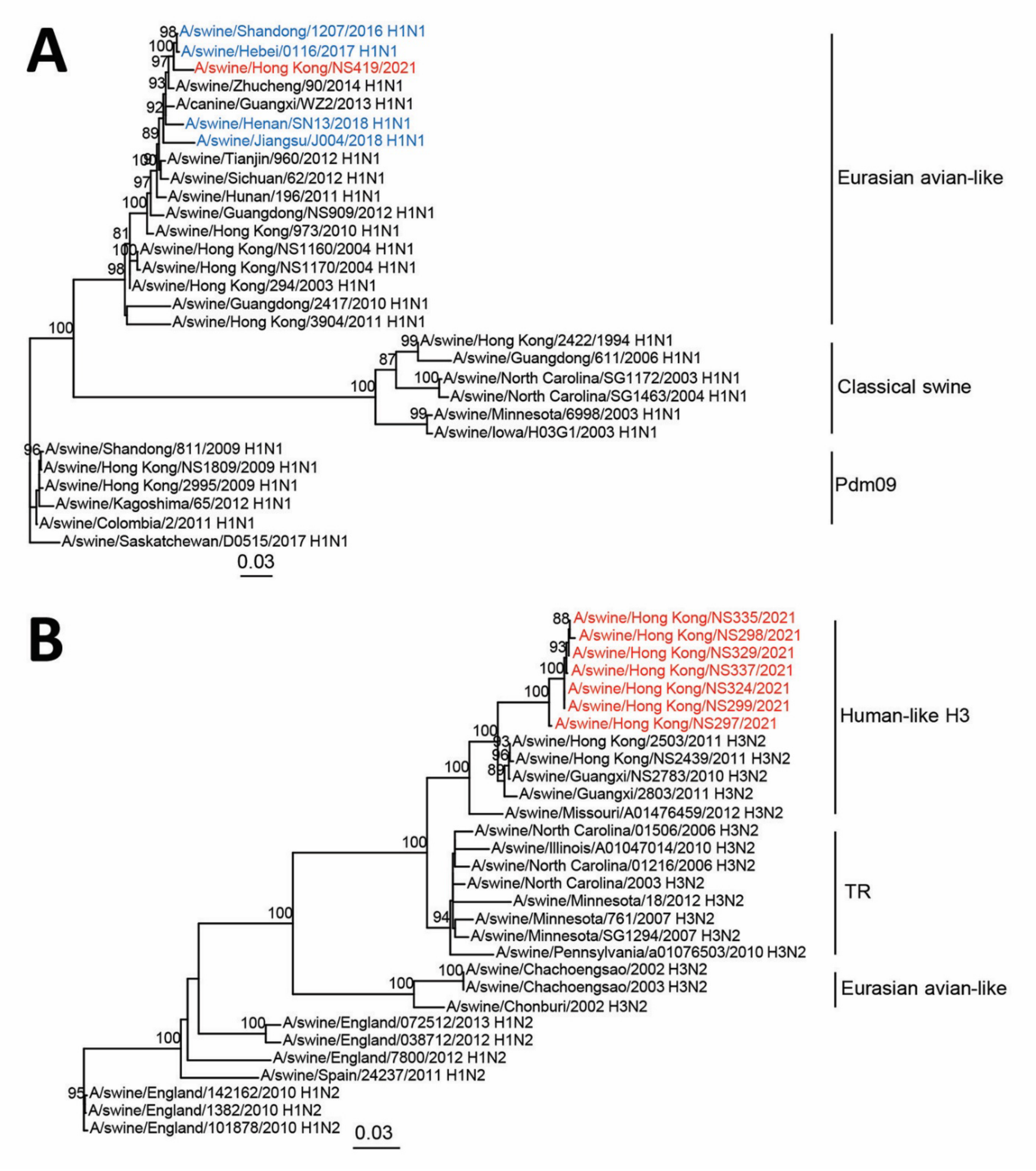

Appendix Figure 2. Phylogenetic tree of N1 (A) or N2 (B) gene sequences. Viral sequences generated from this study (red) and those downloaded from public domains (Appendix Table) were aligned using muscle v3.8 (http://www.drive5.com/muscle/). Virus sequences from G4 swine viruses (Main text) are highlighted in blue. Phylogenetic trees were then constructed by IQ-tree v1.6.12 (http://www.iqtree.org/release/v1.6.12) using the GTR+G model. Major animal viral lineages are as shown. Bootstrap values ≥80% are shown. Scale bar indicates estimated genetic distance.

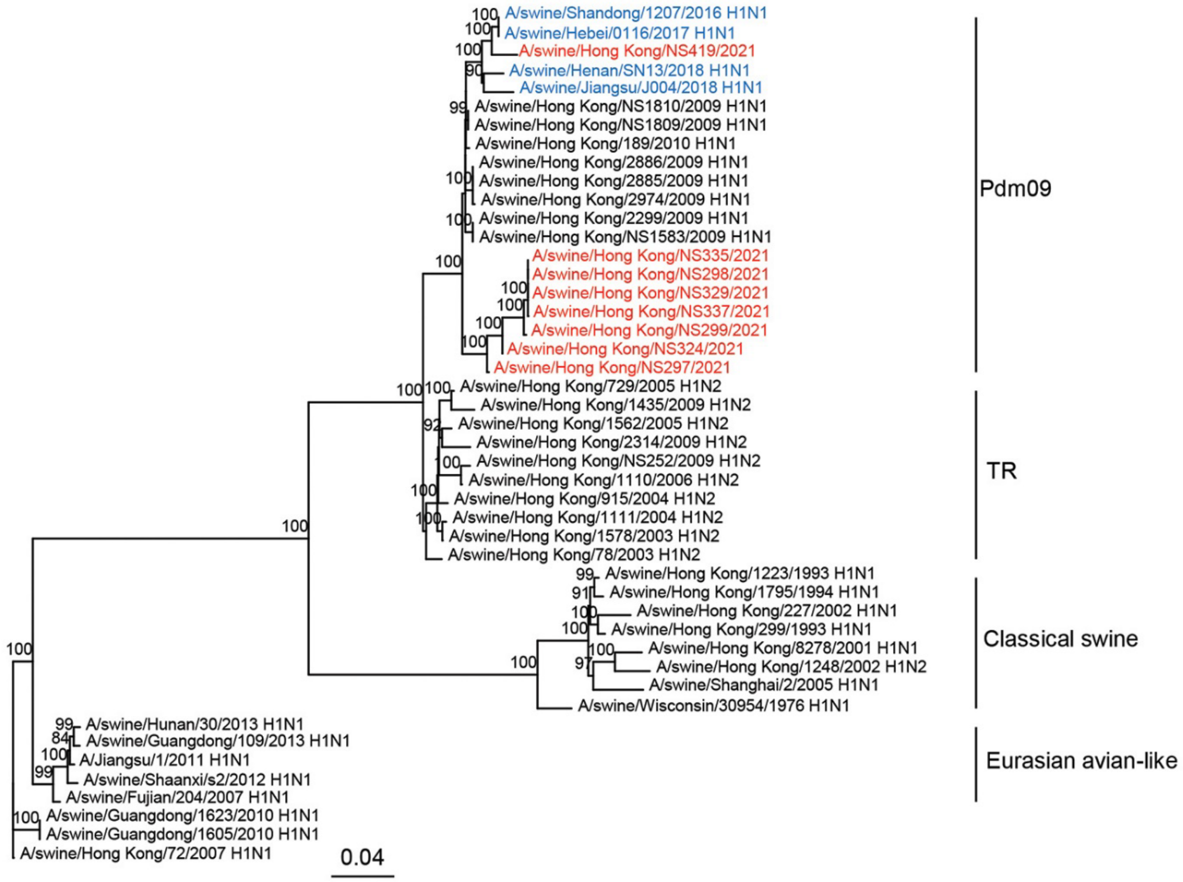

Appendix Figure 3. Phylogenetic tree of PB2 gene sequences. Viral sequences generated from this study (red) and those downloaded from public domains (Appendix Table) were aligned using muscle v3.8 (http://www.drive5.com/muscle/). Virus sequences from G4 swine viruses (Main text) are highlighted in blue. Phylogenetic trees were then constructed by IQ-tree v1.6.12 (http://www.iqtree.org/release/v1.6.12) using the GTR+G model. Major animal viral lineages are as shown. Bootstrap values ≥80% are shown. Scale bar indicates estimated genetic distance.

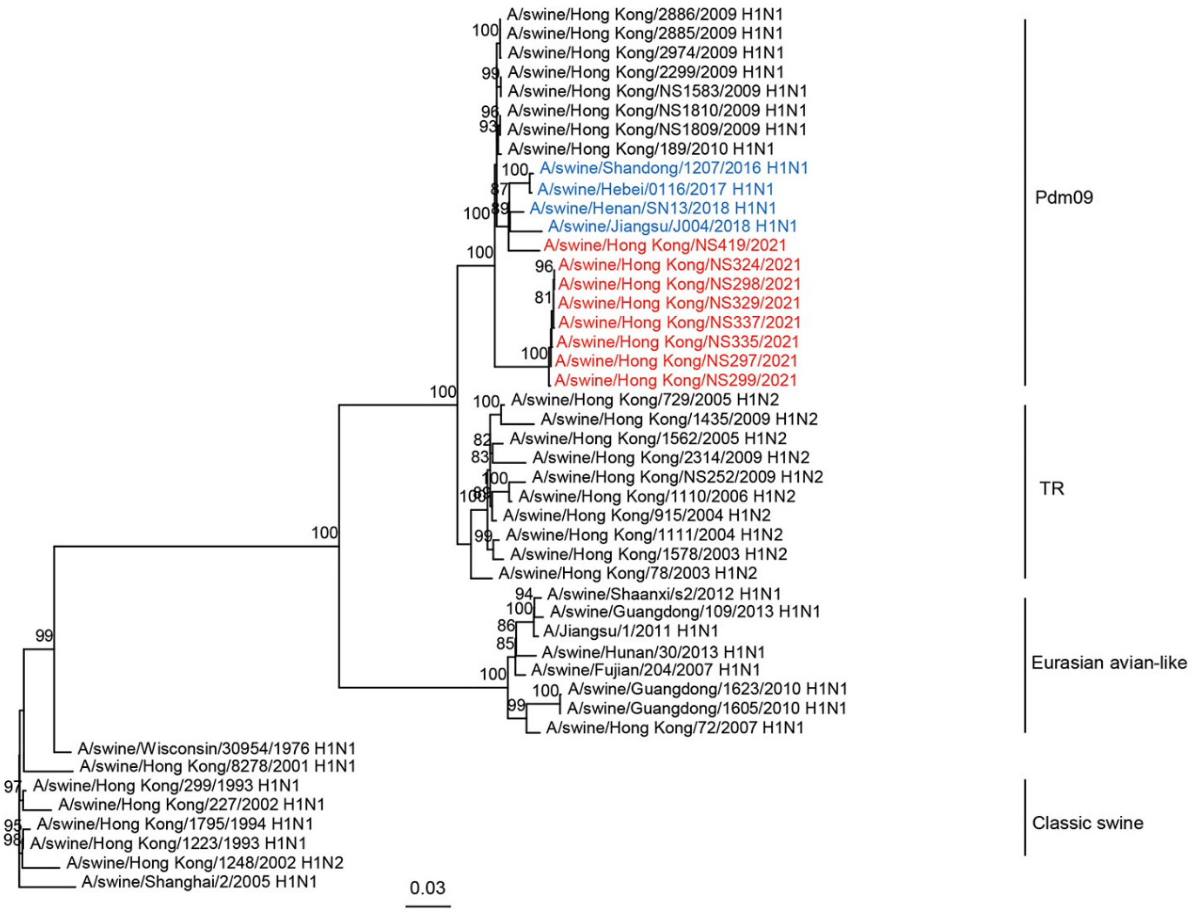

Appendix Figure 4. Phylogenetic tree of PA gene sequences. Viral sequences generated from this study (red) and those downloaded from public domains (Appendix Table) were aligned using muscle v3.8 (http://www.drive5.com/muscle/). Virus sequences from G4 swine viruses (Main text) are highlighted in blue. Phylogenetic trees were then constructed by IQ-tree v1.6.12 (http://www.iqtree.org/release/v1.6.12) using the GTR+G model. Major animal viral lineages are as shown. Bootstrap values ≥80% are shown. Scale bar indicates estimated genetic distance.

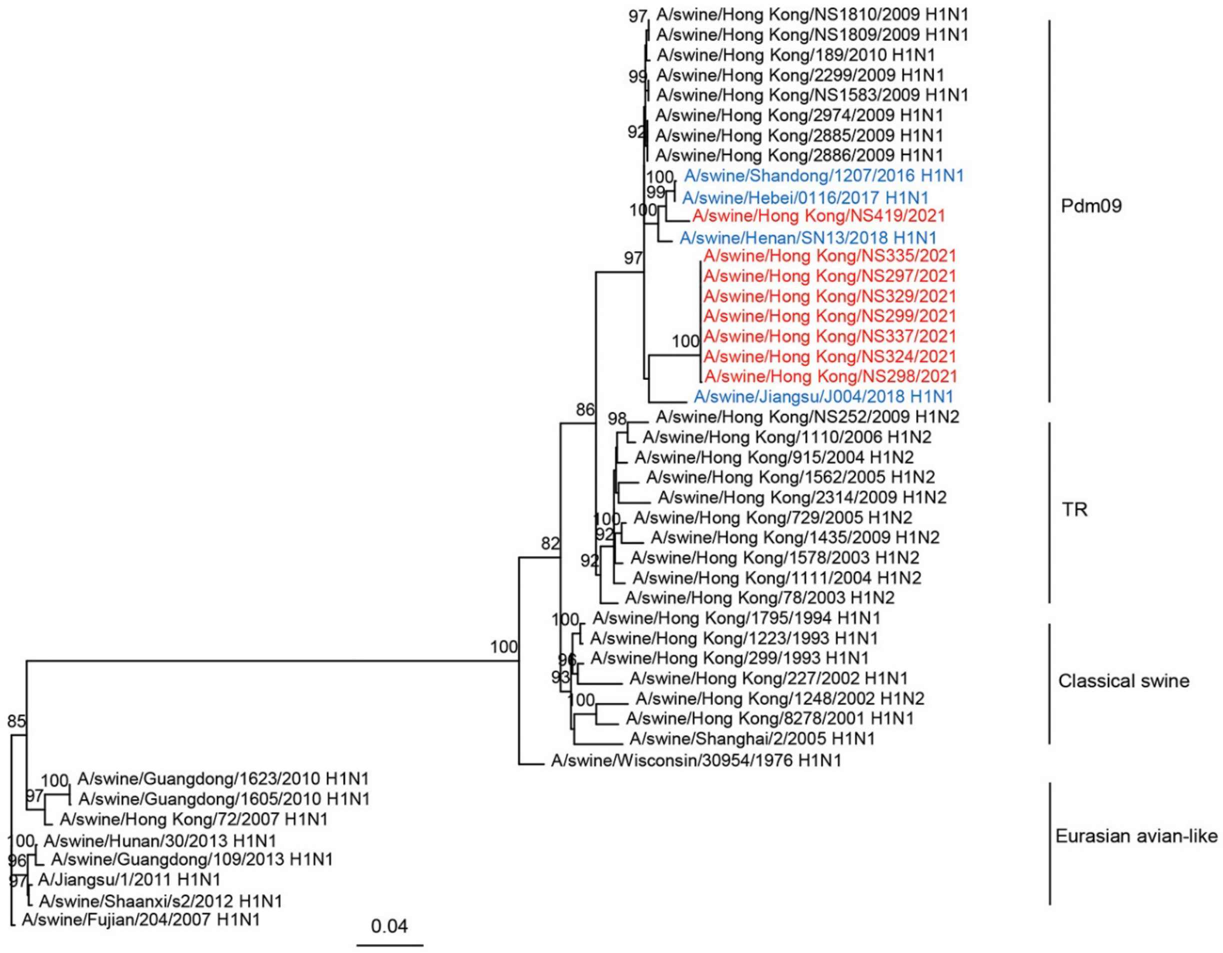

Appendix Figure 5. Phylogenetic tree of M gene sequences. Viral sequences generated from this study (red) and those downloaded from public domains (Appendix Table) were aligned using muscle v3.8 (http://www.drive5.com/muscle/). Virus sequences from G4 swine viruses (Main text) are highlighted in blue. Phylogenetic trees were then constructed by IQ-tree v1.6.12 (http://www.iqtree.org/release/v1.6.12) using the GTR+G model. Major animal viral lineages are as shown. Bootstrap values ≥80% are shown. Scale bar indicates estimated genetic distance.

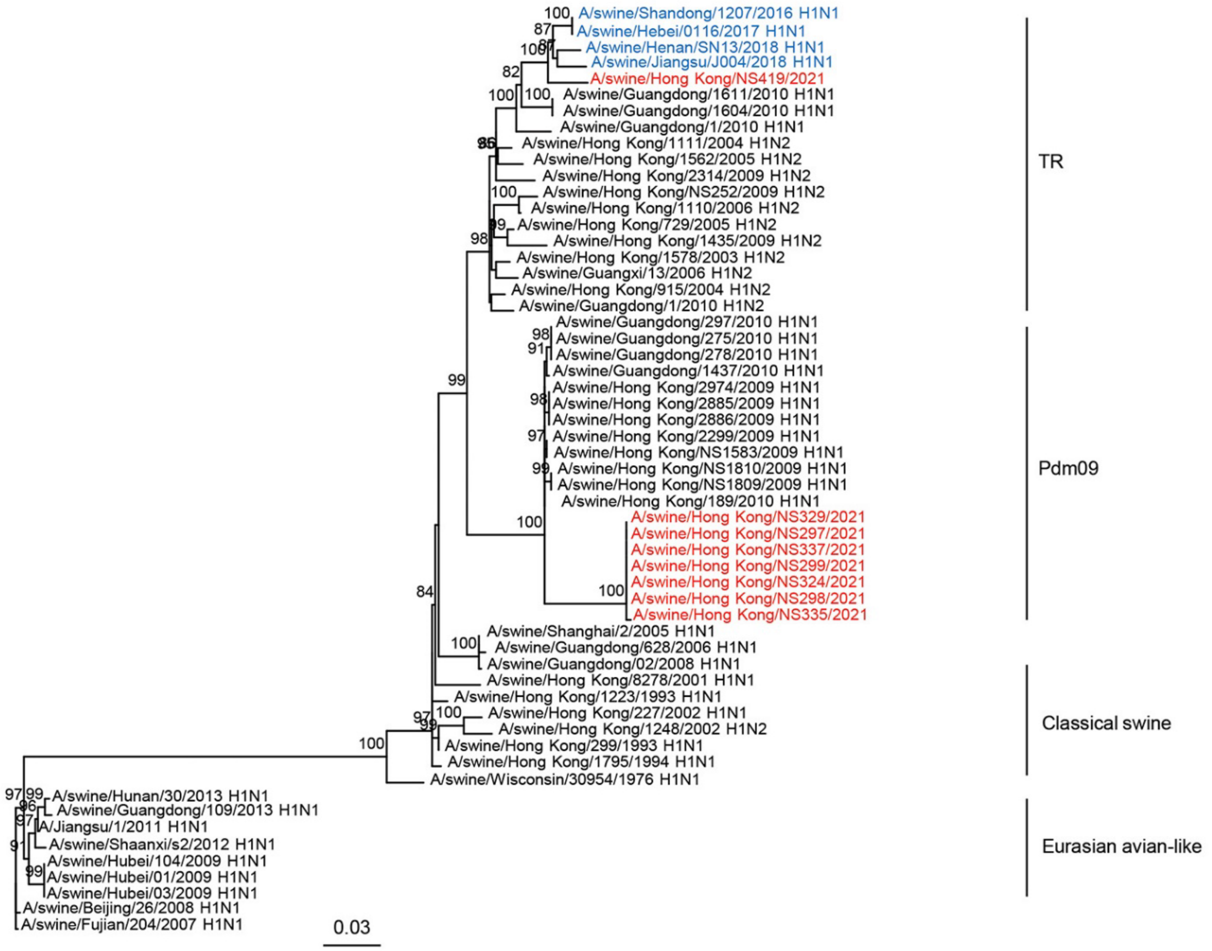

Appendix Figure 6. Phylogenetic tree of NS gene sequences. Viral sequences generated from this study (red) and those downloaded from public domains (Appendix Table) were aligned using muscle v3.8 (http://www.drive5.com/muscle/). Virus sequences from G4 swine viruses (Main text) are highlighted in blue. Phylogenetic trees were then constructed by IQ-tree v1.6.12 (http://www.iqtree.org/release/v1.6.12) using the GTR+G model. Major animal viral lineages are as shown. Bootstrap values ≥80% are shown. Scale bar indicates estimated genetic distance.
